# 168 Community Engagement, One Mile High: Developing a pipeline for training in community-based participatory research for investigators in Colorado – ERRATUM

**DOI:** 10.1017/cts.2023.565

**Published:** 2023-06-14

**Authors:** Kaylee Rivera Gordon, Montelle Tamez, Mary Fisher, Donald E. Nease

The above abstract [1] published with Montelle Tamez’s name spelled incorrectly.

The original abstract has been corrected online to rectify this error.

## References

[ref1] Gordon KR , Tamez M , Fisher M , Nease DE. 168 Community Engagement, One Mile High: Developing a pipeline for training in community-based participatory research for investigators in Colorado. Journal of Clinical and Translational Science 2023; 7(s1): 52. doi: 10.1017/cts.2023.249 PMC10308420 37396817

